# Regulation of *OsmiR156h* through Alternative Polyadenylation Improves Grain Yield in Rice

**DOI:** 10.1371/journal.pone.0126154

**Published:** 2015-05-08

**Authors:** Meng Zhao, Binmei Liu, Kun Wu, Yafeng Ye, Shixia Huang, Shuansuo Wang, Yi Wang, Ruixi Han, Qian Liu, Xiangdong Fu, Yuejin Wu

**Affiliations:** 1 Institute of Technical Biology and Agriculture Engineering, Hefei Institutes of Physical Science, Chinese Academy of Sciences, Hefei, China; 2 The State Key Laboratory of Plant Cell and Chromosome Engineering, Institute of Genetics and Developmental Biology, Chinese Academy of Sciences, Beijing, China; Chinese Academy of Sciences, CHINA

## Abstract

Substantial increases in grain yield of cereal crops are required to feed a growing human population. Here we show that a natural variant of *SEMIDWARF AND HIGH-TILLERING* (*SDT*) increases harvest index and grain productivity in rice. Gain-of-function *sdt* mutation has a shortened polyadenylation tail on the *OsmiR156h* microRNA precursor, which cause the up-regulation of *OsmiR156h*. The plants carrying the semidominant *sdt* allele exhibit reduced plant height, enhanced lodging resistance, increased tiller numbers per plant, and resulting in an increased grain yield. We also show that combining the *sdt* allele with the *OsSPL14^WFP^* allele can be effective in simultaneously improving tillering capacity and panicle branching, thereby leading to higher harvest index and grain yield. Most importantly, pyramiding of the *sdt* allele and the green revolution gene *sd1* enhances grain yield by about 20% in hybrid rice breeding. Our results suggest that the manipulation of the polyadenylation status of *OsmiR156* represents a novel strategy for improving the yield potential of rice over what is currently achievable.

## Introduction

In the cereals, grain productivity is currently heavily dependent on the application of nitrogenous fertilizer. However, over-fertilization with nitrogen causes lodging (stem collapse prior to harvest) with a consequent loss of yield. A rice semi-dwarfing gene, *sd1*, known as the "Green Revolution” gene [1,2], has been extensively used in rice breeding programs over the past 50 years [3]. The continuing growth of the world's population and the limited arable land resources require that grain yield capacity of rice will have to be raised yet further [4–6]. However, grain yield loss due to lodging remains a problem in many high-yielding varieties carrying the Green Revolution *sd1* gene [7], and the predominant use of this gene by famers and breeders is due to the lack of other useful semi-dwarfing genes, leading to narrowing down the genetic base of modern cultivars [8, 9].

It has been shown that *miR156* involves in many plant growth and developmental processes [10]. The SQUAMOSA-PROMOTER BINDING PROTEIN-LIKE (SPL) transcription factor is the direct targets of *miR156* in various plant species [10,11], which regulates flowering time, plastochron length, trichome patterning, tiller number and panicle branching [12–16]. In particular, the natural variants of the *OsSPL14* gene (e.g. *IPA* and *FWP*) play the important role in the regulation of plant architecture and panicle branching in rice [14,15], where *OsSPL16* has been reported to be involved in the control of grain size, shape and quality in rice [17]. There is a well-established negative correlation between tillering capacity and panicle branching, meaning that achieving a simultaneous genetic gain in both tiller numbers per plant and grain numbers per panicle represents a major challenge for rice breeders. Here, we show that we identified a natural variant of *SEMIDWARF AND HIGH-TILLERING* (*SDT*), which reduces plant height, increases tiller numbers per plant and enhances grain yield in rice. The further positional cloning and genetic complementation analysis demonstrate that the *sdt* allele is involved an alternative polyadenylation of the *OsmiR156h* microRNA precursor, and the up-regulation of *OsmiR156h* promotes tillering capacity and enhances grain yield.

## Results

### A natural allelic variant of *sdt* influences plant height and tillering

**Fig 1:**
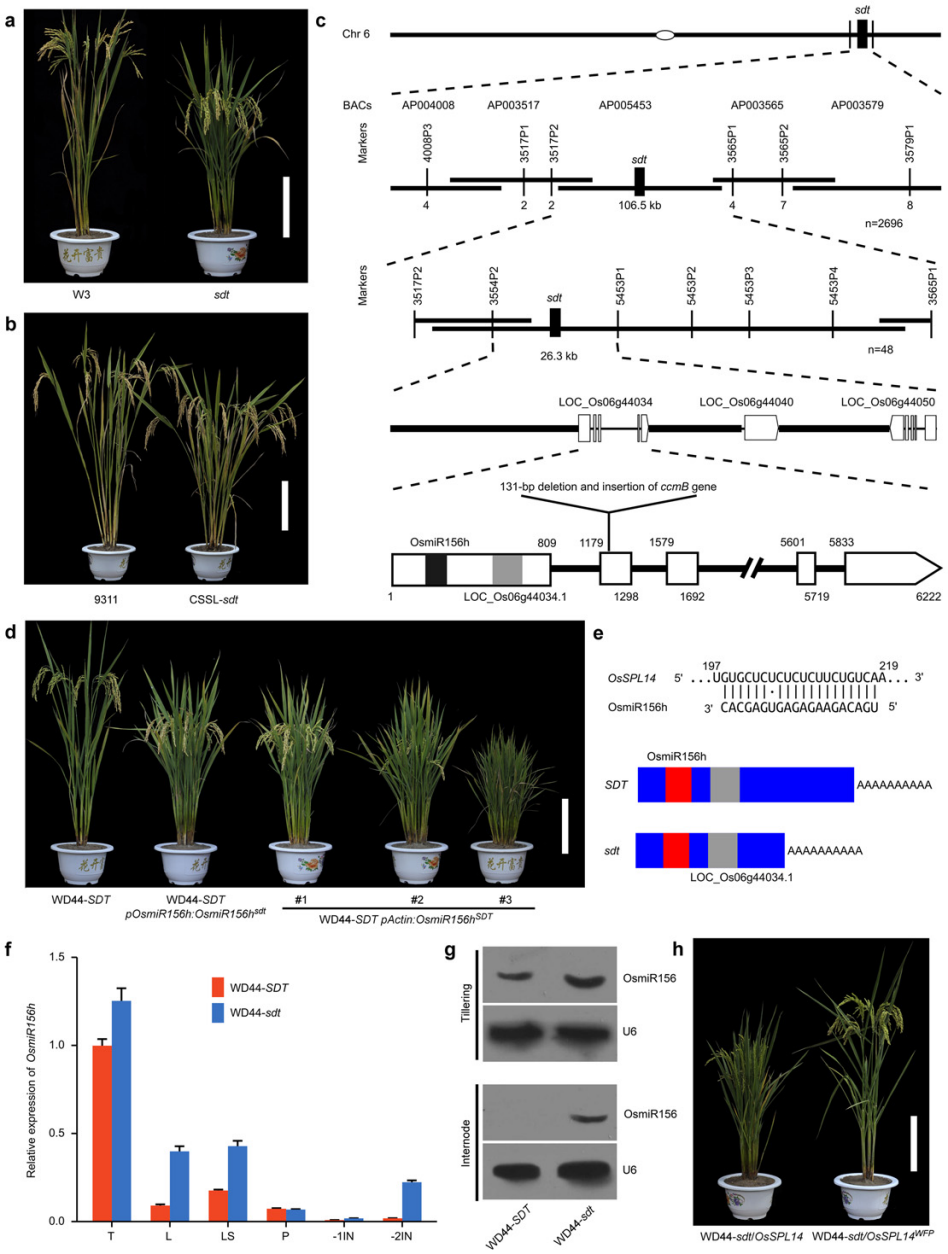
Positional cloning of *sdt*. (**a**) The phenotype of parental plants. Scale bar: 20 cm. (**b**) Mature plant appearance of CSSLs. Scale bar: 20 cm. (**c**) In the fine-scale map, the QTL falls in the candidate region between markers 3517P2 and 3565P2 using 2,696 BC_1_F_2_ segregants, and the progeny test of homozygous recombinant individuals further narrowed to a ~26.3 kb segment flanked by markers 3545P2 and 5453P1. The numbers below the line indicate the number of recombinants between *sdt* and the marker shown. Open bars represent the exons of the predicted genes, and filled bars represent putative transcripts for, respectively, LOC_Os06g44034.1 and *OsmiR156h* precursor. (**d**) Mature plant phenotype of the transgenic plants expressing *OsmiR156h*. Scale bar: 20 mm. (**e**) Allelic variation of 3’-UTR in the *SDT* locus. Red and gray rectangles represent the transcripts for, respectively, *OsmiR156h* stem-loop sequence and LOC_Os06g44034.1. (**f**) Expression of *OsmiR156h* in WD44-*SDT* and NIL-*sdt*. Expression levels of *OsmiR156h* were analyzed by stem-loop real-time RT-PCR. T: developing tiller buds; L: flag leaf; LS: leaf sheath; P: young panicle. -1IN: the first internode (the uppermost internode); -2IN: the second internode. Expression levels are expressed as the relative copies per 1000 copies of rice *actin3*. Data given as mean ± SE (n = 3). (**g**) Northern blot analysis of *OsmiR156* levels in the NILs plants. Blots of total RNA extracted from young tillers of 55-day-old plants and the second internodes of 80-day-old plants, respectively. U6 was used as a loading control for *OsmiR156*. (**h**) The phenotypes of the WD44-*sdt*/*OsSPL14* ^*WFP*^ and WD44-*sdt*/*OsSPL14* plants. Scale bar: 20 mm.

To investigate novel approaches for improving lodging resistance to support heavy panicles under the high nitrogen conditions, a spontaneous rice *semidwarf and high-tillering* (*sdt*) mutant was selected based on different responses to nitrogen fertilization. The *sdt* mutant plants displayed the reduced plant height and an increased number of tiller per plant when compared with a typical *indica* variety Wan3 (W3) (Fig 1a). Among a set of chromosome segment substitution lines (CSSLs) derived from the cross between the *sdt* mutant (the donor parent) and the *indica* variety 9311 (the recurrent parent), one CSSL line exhibited the similar semidwarf and high-tillering phenotypes as the *sdt* mutant, which we named as CSSL-*sdt* (Fig 1b). A subsequent genetic analysis of the selfed progeny of the backcross (CSSL-*sdt* ×9311)×9311 identified a major quantitative trait locus *qsdt* responsible for semidwarfism and high tillering that was mapped to the long arm of chromosome 6 (Fig 1c). The phenotype of BC_2_F_2_ segregants heterozygous at this locus was intermediate between that of the two alternative homozygotes (S1 Fig), which indicated that the *sdt* allele is semidominant.

### Positional cloning of *sdt*

A high resolution genetic map based on 2,696 BC_1_F_2_ individuals bred from the cross CSSL-*sdt* × W3 narrowed the genomic location of *qsdt* to a ~106.5-kb region defined by molecular markers 3517P2 and 3565P2. The progeny test of homozygous recombinant individuals further narrowed to a ~26.3-kb segment flanked by molecular markers 3545P2 and 5453P1, a segment which contains three predicted genes (Fig 1c). A sequence comparison of parental copies of the three putative genes showed that there wasn’t any single nucleotide polymorphism (SNP) with respect to either LOC_Os06g44040 or LOC_Os06g44050, but that a 131-bp segment present in the second exon of LOC_Os06g44034 of the W3 allele had been replaced in CSSL-*sdt* by an inverted fragment of the mitochondrial gene *ccmB* (cytochrome c biogenesis B) [18] (Fig 1c and S2 Fig). LOC_Os06g44034 contains five exons and four introns, the first exon encodes an *OsmiR156h* microRNA precursor and a protein-coding transcript (LOC_Os06g44034.1) (Fig 1c), while the other exons generate a long 3'-UTR. Quantitative RT-PCR analysis showed that the increased transcription level of LOC_Os06g44034 was dependent on the presence of the insertion polymorphism (S3 Fig).

### *OsmiR156h* is associated with semidwarf and high-tillering phenotype

To perform genetic complementation analysis, we developed a near-isogenic line, WD44-*sdt*, which is homozygous for the *sdt* mutant allele in the high-yielding *japonica* variety Wandao44 background, whereas WD44-*SDT* is homozygous for the Wandao44 *SDT* allele. The transgenic WD44-*SDT* plants, in which the LOC_Os06g44034.1 cDNA from WD44-*sdt* was constitutively overexpressed, had no visible alteration in either plant height or the number of tillers per plant (S4 Fig). However, the transgenic WD44-*SDT* plants expressing the WD44-*sdt OsmiR156h* sequence under the control of its native promoter exhibited reduced plant height and increased tiller numbers when compared with non-transgenic WD44-*SDT* plants (Fig 1d). The transgenic WD44-*SDT* plants carrying the transgene construct harbored the WD44-*SDT OsmiR156h* also displayed increased tiller numbers and dwarfed phenotypes, with transcript abundance of *OsmiR156h* being positively correlated with the extent of phenotypic change [10] (Fig 1d and S5 Fig). In addition, we found that down-regulation of *OsSPL14* (*SQUAMOSA PROMOTER BINDING PROTEIN- LIKE14*), which is a direct target of *OsmiR156h* [14,15,19] (Fig 1e), led to increases in tiller numbers per plant and reduction of plant stature (S6 Fig). These results suggested that *OsmiR156h*-*OsSPL14* regulatory module plays an important role in the regulation of tillering capacity. The *miR156-SPL* module has been suggested to be conserved in land plant evolution [15,20–22]. We also found that the transgenic wheat plants constitutively expressing *OsmiR156h* exhibited increased tiller numbers and the dwarf stature (S7 Fig). Thus, *OsmiR156h* is the sequence responsible for the *sdt* QTL.

### Alternative polyadenylation of *OsmiR156h* contributes to the *sdt* phenotype

A 3’-RACE analysis targeting the *OsmiR156h* precursor was performed to investigate whether *OsmiR156h* expression was affected by alternative 3’-UTR polyadenylation of *OsmiR156h* precursor transcript [23,24]. The predicted 1,552-bp fragment was amplifiable from WD44-*SDT*, which has the same exon borders as the annotated exons. However, a transcript shortened at the 3'-UTR was present in WD44-*sdt* (Fig 1e and S8 Fig). The shortening of 3’-UTR was due to the loss of a 643-nt segment which corresponded to the sequences of exons 3 through 5, together with an additional 76-nt upstream of the poly(A) tail. The shortened 3'-UTR included a poly(A) signal sequence (AATAAA) located 54-nt upstream of the poly(A) itself (S9 Fig). *OsmiR156h* precursor transcripts were detectable in various organs in rice. A higher abundance was noted in developing tiller buds, while a much lower abundance was detected in the leaf, leaf sheath, culm and young panicle (Fig 1f). Nevertheless, the abundance of both the *OsmiR156h* precursor and the mature transcripts were higher in WD44-*sdt* than in WD44-*SDT* (Fig 1f and 1g).

The *OsSPL* genes are targeted by *OsmiR156* [14,15,17], the transcriptional levels of a number of such genes were compared between WD44-*sdt* and WD44-*SDT*. We found that *OsmiR156h*-targeted *OsSPL* genes proved to be down-regulated in various parts of WD44-*sdt* plants, such as *OsSPL14* [14,15] and *OsSPL16* [17] (S10 Fig). It is known that the *OsSLP14* ^*WFP*^ allele produces a higher abundance of *OsSPL14* transcript [15]. When the *OsSLP14* ^*WFP*^ allele was introduced into WD44-*sdt*, the forming phenotype was similar to that of WD44-*SDT* (Fig 1h). Taken together, these genetic results indicated that the up-regulation of *OsmiR156h* through the alternative 3’-UTR polyadenylation produces the *sdt* phenotype.

### Introduction of the *sdt* allele into the elite rice variety improves grain yield

**Fig 2:**
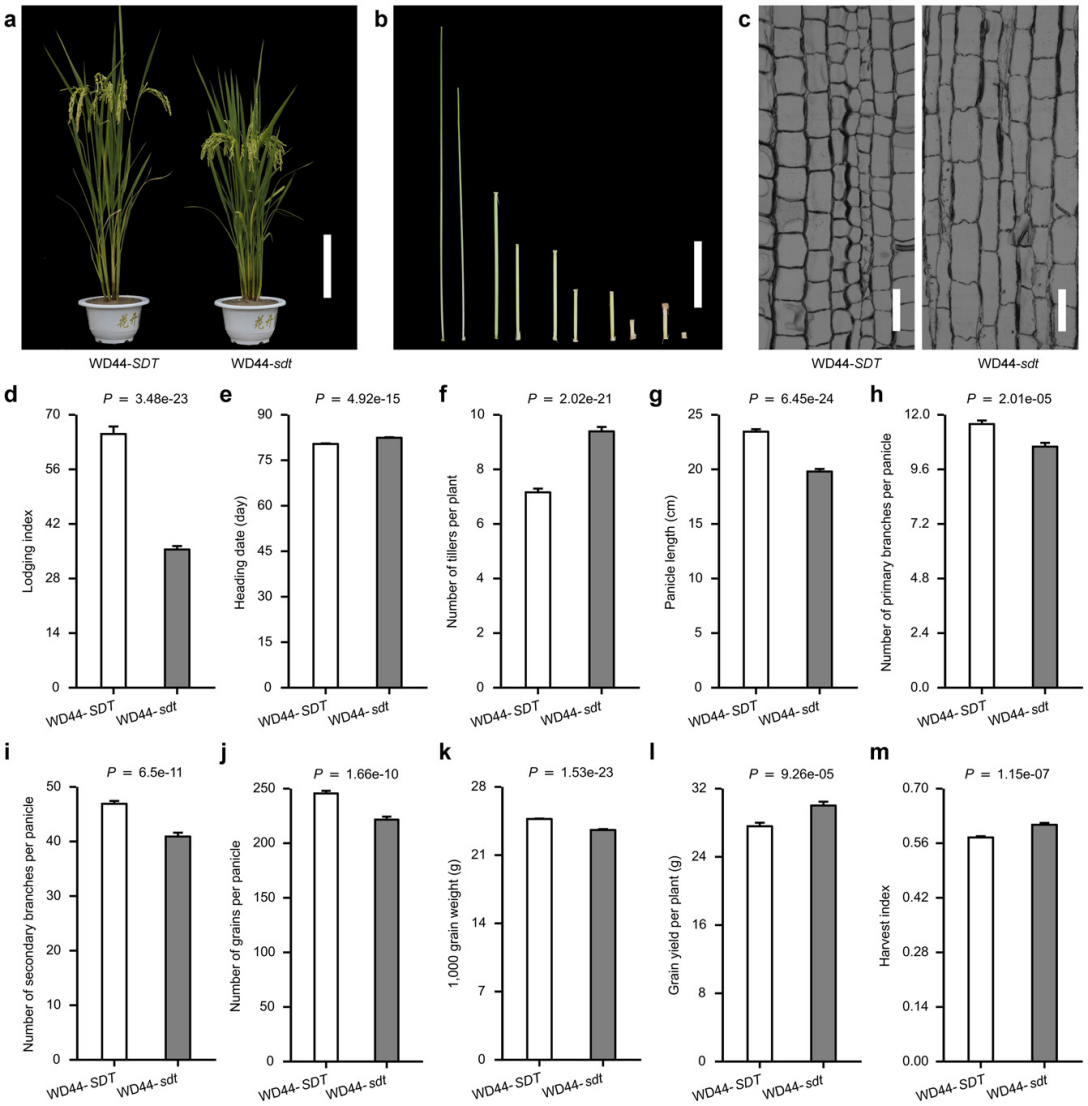
The contrasting phenotype of WD44-*SDT* and WD44-*sdt*. (**a**) Mature plant appearance. Scale bar: 20 cm. (**b**) Culm internode length. Scale bar: 20 cm. (**c**) The effect of the *sdt* allele on cell proliferation: longitudinal sections of the uppermost internode. Scale bar: 0.1 mm. (**d**) Lodging index [30] (**e**) Heading date. (**f**) Tiller numbers per plant. (**g**) Panicle length. (**h**) Number of primary branches per panicle. (**i**) Number of secondary branches per panicle. (**j**) Number of grains per panicle. (**k**) 1,000 grain weight. (**l**) Grain yield per plant. (**m**) Harvest index. All phenotypic data were measured from the plants in randomized complete block design with three replications, which were grown with a distance of 20 × 20 cm in paddies under normal cultivation conditions. Data represented mean ± SE (n = 120). A Student’s *t*-test was used to generate the *P* values.

WD44-*sdt* plants were semidwarf in stature (Fig 2a and 2b), but lacked some of the negative pleiotropic effects when compared to those transgenic plants associated with the constitutive expression of *OsmiR156*, such as the strongly reduced panicle size and delayed heading date (Fig 1d). To investigate effect of the *sdt* allele on grain yield, the field performance of WD44-*SDT* and WD44-*sdt* was compared under normal cultivation conditions [25]. The two lines did not differ from one another with respect to their heading date (Fig 2e), whereas the number of tillers per plant of WD44-*sdt* was about 30% greater than that of WD44-*SDT* (Fig 2f). WD44-*sdt* plants produced shorter panicles and a lower number of both primary and secondary branches, which together resulted in the reduction in the number of grains formed per panicle as well as the production of smaller grains (Fig 2g–2k). However, the overall grain yield per plant of WD44-*sdt* was about 9% above that of WD44-*SDT* (Fig 2l), and its harvest index (ratio of grain weight to whole plant above ground biomass) was about 5% higher (Fig 2m). Thus, the *sdt* allele has the potential to both improve harvest index and the overall grain yield in rice. We also found that the longitudinal sections of the uppermost internode showed WD44-*sdt* internode cells were longer than those in WD44-*SDT*, but the length of each internode was less in WD-*sdt* than in WD44-*SDT* (Fig 2c). This outcome suggested that *sdt* functions as a negative regulator of cell proliferation in the stem, but it enhanced lodging resistance [26] (Fig 2d).

### Combining *sdt* with *OsSPL14* ^*WFP*^ can be effective in simultaneously improving tillering capacity and panicle branching

There is a well-established negative correlation between tillering capacity and panicle branching, meaning that achieving a simultaneous genetic gain in both tiller numbers per plant and grain numbers per panicle represents a major challenge for rice breeders [27,28]. Both the *OsSLP14* ^*WFP*^ and *OsSPL14* ^*ipa*^ alleles have been shown to promote panicle branching [14,15], but the up-regulation of *OsSPL14* substantially reduces the number of tiller per plant [29] (S11 Fig). The ability of the *sdt* allele to compensate for this negative effect was tested by breeding the near-isogenic line NIL-*OsSLP14* ^*WFP*^ */sdt* in a background of the *indica* variety NP174. Field-grown NIL-*OsSLP14* ^*WFP*^/*sdt* plants were shorter, and produced about 55% more tillering than that formed by NIL-*OsSLP14* ^*WFP*^/*SDT* plants (Table 1 and S12 Fig). Although NIL-*OsSLP14* ^*WFP*^/*sdt* plants set fewer grains per panicle than that formed by NIL-*OsSLP14* ^*WFP*^/*SDT* plants, the combination of *sdt* with *OsSLP14* ^*WFP*^ increased lodging resistance and overall grain yield by about 20%, and its harvest index was about 25% higher than that of plants carrying a single mutated allele (Table 1).

**Table 1:** Comparison of grain yield performance between NIL-*OsSPL14* ^*WFP*^ */SDT* and NIL-*OsSPL14* ^*WFP*^ */SDT* plants.

Traits	NIL-*OsSPL14* ^*WFP*^/*SDT*	NIL-*OsSPL14* ^*WFP*^/*sdt*
**Number of tillers per plant**	7.83 ± 0.09	12.24 ± 0.14
**Number of primary rachis branches per panicle**	16.40 ± 0.14	19.40 ± 0.32
**Number of secondary rachis branches per panicle**	83.60 ± 1.07	60.25 ± 0.86
**Number of grains per panicle**	274.69 ± 3.55	194.04 ± 1.45
**1,000 grain weight (g)**	23.74 ± 0.24	26.56 ± 0.47
**Lodging index**	149.63 ± 3.65	63.62 ± 1.86
**Grain yield per plant (g)**	34.95 ± 0.59	42.08 ± 0.34
**Harvest index**	0.48 ± 0.01	0.60 ± 0.01

All phenotypic data were measured from the NILs plants in randomized complete block design with three replications, which were grown with a distance of 20 × 20 cm in paddies under normal cultivation conditions. Data given as mean ± SE (n = 120).

### Pyramiding of the *sdt* and *sd1* alleles improves grain yield in hybrid rice breeding

**Fig 3:**
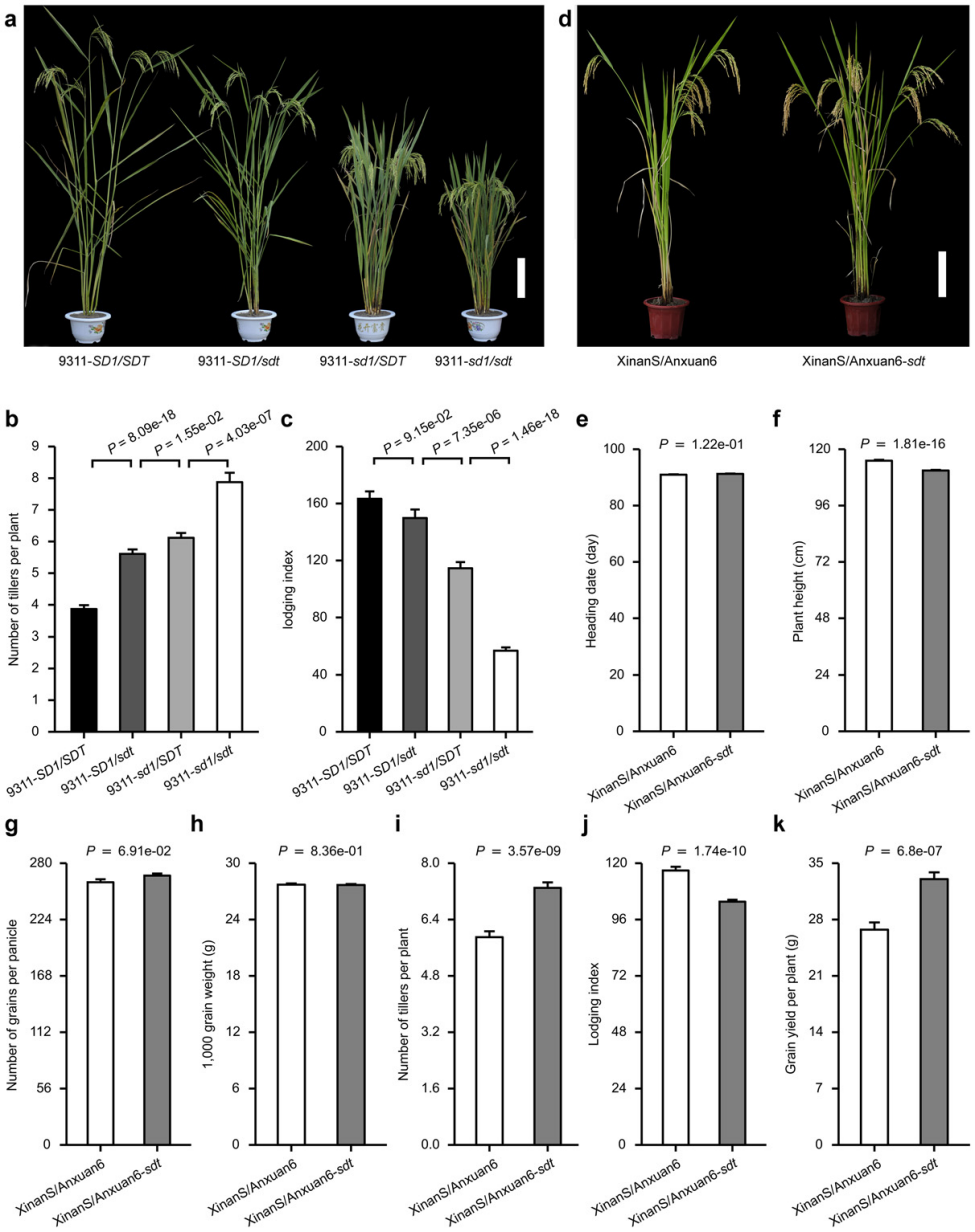
Pyramiding of *sdt* and *sd1* alleles improves lodging resistance and grain yield. (**a**) Mature plant phenotype of four near-isogenic lines in a background of the *indica* variety 9311. Scale bar: 20 cm. (**b**) The allelic combination of *sdt* and *sd1* increases tiller numbers per plant. Data represented mean ± SE (n = 60). (**c**) Combining *sdt* with *sd1* enhances lodging resistance [30]. Data represented mean ± SE (n = 60). (**d**-**j**) The contrasting phenotype and grain yield of F_1_ hybrid plants. (**d**) Mature plant appearance. Scale bar, 20 cm; (**e**) Plant height; (**f**) Number of grains per panicle; (**g**) 1,000 grain weight; (**h**) Number of tillers per plant; (**i**) Lodging index; (**j**) Grain yield per plant. Data were measured from the plants in randomized complete block design with three replications, which were grown with a distance of 20 × 20 cm in paddies under normal cultivation conditions. Data represented mean ± SE (n = 120). A Student’s *t*-test was used to generate the *P* values.

The Green Revolution rice semi-dwarfing gene (*sd1*) has been the backbone of the major increase in grain yield achieved over the past 50 years. The interaction between *sd1* and *sdt* was explored by comparing a set of four near-isogenic lines in a background of the high-yielding *indica* variety 9311 (Fig 3a). Short stature and high tillering were conferred by the presence of either *sd1* or *sdt*, but plants carrying both *sd1* and *sdt* were shorter, tillered more profusely and were less susceptible to lodging than those plants carrying only single semi-dwarfing gene (Fig 3a–3c). We next evaluated whether pyramiding of *sdt* and *sd1* alleles could be used to improve grain yield and lodging resistance, particularly in super hybrid rice breeding [30]. A two-line hybrid rice combination XinanS/Anxuan6 (Xinliangyou 6) developed from the cross between XinanS (the photo-thermo sensitive genic male sterile line) and Anxuan6 (the restorer line carrying the *sd1* allele) has been predominately cultivated in Yangtze River area in China, the utility of a NIL line Anxuan6-*sdt* carrying both *sd1* and *sdt* was investigated (Fig 3d). No perceptible effect on heading date, plant height, and the number of grains per panicle or grain weight were evident between the hybrid combination XinanS/Anxuan6 and XinanS/Anxuan6-*sdt* (Fig 3e–3g). However, the presence of the *sdt* allele increased tiller numbers per plant and lodging resistance (Fig 3h and 3i), and improved gain yield by 20% (Fig 3j). Therefore, the manipulation of *OsmiR156* expression through alternative polyadenylation represents a useful strategy for breaking the unfavorable correlation between tillering capacity and panicle branching, which in turn offers a route to higher grain yield over that of existing high-yielding inbred and hybrid rice varieties.

## Discussion

The gain-of-function rice *sdt* mutant has increased tiller number, which is the key factor accounting for the improvement of grain yield per plant, while the number of grain number per panicle and 1,000-grain weight are all reduced. Although the WD44-*sdt* internode cells were longer than those in WD44-*SDT*, the length of each internode was less in WD44-*sdt* than that in WD44-*SDT*, suggesting that *sdt* functions as a negative regulator of cell proliferation in the stem, and consequently resulting in enhanced lodging resistance. The previous studies have shown that mutated *IPA1* (*Ideal Plant Architecture 1*) allele of *OsSPL14* can promote panicle branching and reduce the formation of tillering, which defines the "ideal plant architecture" phenotype and potentially improves grain yield [14,15]. Although more panicle branches have better potential to increase the yield, plants with that trait tend to have low seed setting percentage since nutrition is limited. In fact, the total panicle number of rice population is the guarantee of high yield in rice production, those varieties with more tillers can realize the stable grain yield easily under the ambient environmental conditions.

The over-expression of the *miR156*-insensitive *ipa1* allele of the *OsSPL14* gene led to an ideal plant architecture in rice by reducing the number of tillers per plant and increasing the number of grain per panicle [14,15]. On contrast, Xie *at al*. showed that over-expression of *OsmiR156* exhibited higher branches, but it caused the reduction of grain numbers [10]. We also found that over-expression of *OsmiR156h* under the rice *Actin* promoter does not lead to favorable phenotypes because the transgenic plants produced too many tillering. We also found that higher expression of *OsSPL14* also resulted in the decreases in panicle branching (S11 Fig), suggesting that the up-regulation of *miR156* could lead to different rice plant phenotypes: promote or repress panicle branching. These results indicates that the moderate transcriptional level or the expression patterns of *OsmiR156h* is critical for modulating the balance between panicle branching and tillering capacity and improving grain yield in rice.

The *sdt* allele is involved in shortening the polyadenylation tail of *OsmiR156h* microRNA precursor. As described above, a segment located at the second exon of LOC_Os06g44034 was replaced by an inverted fragment of *ccmB* in the *sdt* mutant, and this insertion mutation causes shortened 3'UTR of the *OsmiR156h* precursor transcript, which in turn increases the abundance of *miR156h*. Most importantly, pyramiding of *sdt* and *OsSPL14* ^*WFP*^ elite alleles overcame the reduction in tiller numbers associated with overexpression of *OsSPL14*, and increased the overall grain yield by about 20%. This result indicated that combining *sdt* with *OsSPL14* ^*WFP*^ can be effective in simultaneously improving tillering capacity and panicle branching, leading to higher overall grain yield. Taken together, the manipulation of the polyadenylation status of *OsmiR156* represents a novel strategy to coordinately regulate the balance between panicle branching and tiller numbers in rice. The *miR156-SPL* regulatory module will be useful for farmers and breeders to improve grain yield potential of rice over what is currently achievable.

## Materials and Methods

### Plant materials

The CSSL population was generated by single seed descent from the cross *sdt* mutant × 9311, and the NILs by backcrossing CSSL-*sdt* with either WD44, NP174, 9311 or Anxuan6. Paddy-grown rice plants were raised during the standard rice growing season at an experimental station in Institute of Technical Biology and Agriculture Engineering, Hefei Institutes of Physical Science, Chinese Academy of Sciences (Anhui Province).

### Mapping of *sdt*

Fine mapping was based on a set of 2696 BC_1_F_2_ progeny bred from the backcross (CSSL-*sdt* × W3) × W3. The sequences of the three predicted genes in the candidate region were compared among the mapping parents. Markers used for genotyping are listed in S1 Table.

### Transgene constructs

The full-length LOC_Os06g44034.1 cDNA was amplified from leaf tissues of WD44-*sdt* plants, and then cloned into *pActin*::*ocs* vector. Constructs driving the constitutive expression of the putative *OsmiR156h* precursor were generated by introducing the genomic sequence containing the *OsmiR156h* precursor into *pCAMBIA2300* vector [17]. Transgenic rice and wheat plants were produced by *Agrobacterium*-mediated transformation [17,31]. Relevant primer sequences and vectors are showed in S2 Table and S13 Fig.

### qRT-PCR analysis

Total RNA was extracted from various parts of the rice plant using the TRIzol reagent (Invitrogen), converted to cDNA and used as a template for real-time PCR as described elsewhere [32]. Each experiment was repeated at least three times, with the rice *actin3* gene used as a reference sequence. Relevant PCR primer sequences are given in S3 Table.

### 3’-RACE

3’-RACE PCR was carried out using the 3’-RACE Kit (Takara D314) following the manufacturer's instructions. The PCR relied on the nested adaptor primer and specific primers for the first exon of LOC_Os06g44034. Relevant PCR primer sequences are given in S4 Table.

### RNA blots

Total RNA was separated by electrophoresis through a 19% denaturing polyacrylamide gels, and transferred to a Hybond N+ nylon membrane (Amersham Bioscience, GE Healthcare) as described previously [33]. After hybridization at 40°C in ULTRAhyb-oligo hybridization buffer (Ambion, Austin, TX) with a biotin-labeled (Invitrogen) mixed probe (OsmiR156 and U6), the membranes were washed twice at 40°C in 2×SSC and 0.5% SDS for 30 minutes before scanning.

## Supporting Information

S1 Fig Effect of the semidominant *sdt* allele on plant height. Segregation of the BC_2_F_2_ population derived from the backcross between the selected BC_1_F_2_ progeny carrying the *sdt* allele and 9311 plant. Comparisons of plant height among homozygotes of the *sdt* allele, heterozygotes of the *sdt* and the *SDT* allele, and homozygotes of the *SDT* allele. Data represented as mean ± SE (n = 30).
(TIF)

S2 Fig Sequence alignment of the LOC_Os06g44034 locus. Allelic variation at the *sdt* locus, including a one-nucleotide substitution (g.1205C>G), a 131-bp deletion (g.1280_1401del) and an insertion of DNA fragment of the mitochondrial gene *ccmB*. The numbers indicate the position of the genomic sequence counted from the transcription start site of LOC_Os06g44034, and the lines above DNA sequences represent the location of the exons.
(TIF)

S3 Fig Comparison of relative abundance of transcripts between CSSL-*sdt* and its recurrent parent. The transcriptional levels were determined by qRT-PCR using young leaf tissues. Expression levels are expressed as the relative copies per 1000 copies of rice *actin3*. Data given as mean ± SE (n = 3).
(TIF)

S4 Fig Phenotype of transgenic rice plants constitutively expressing LOC_Os06g44034.1. Mature plant appearance of the transgenic WD44-*SDT* plants overexpressing LOC_Os06g44034.1 under the control of rice *Actin* promoter. Scale bar: 20 cm.
(TIF)

S5 Fig Phenotypic characterization of the transgenic WD44-*SDT* plants overexpressing *OsmiR156h*. (**a**) The transcriptional levels of *OsmiR156h* were determined by qRT-PCR. Expression levels are expressed as the relative copies per 1000 copies of rice *actin3*. Data given as mean ± SE (n = 3). (**b**) Plant height. (**c**) Tiller numbers per plant. The transgenic plants were shown in Fig 1d.
(TIF)

S6 Fig Phenotypic characterization of the transgenic rice plants carrying an RNAi-*OsSPL14* construct. (**a**) Mature plant appearance. Scale bar: 20 cm. (**b**) Plant height. (**c**) Tiller numbers per plant. Data given as mean ± SE (n = 10). A Student’s *t*-test was used to generate the *P* values.
(TIF)

S7 Fig The phenotype of the transgenic wheat plants constitutively expressing *OsmiR156h*. A winter wheat variety KN199 was used to generate the transgenic plants carrying the *p35S*::*OsmiR156h* ^*sdt*^ construct. Scale bar: 20 cm.
(TIF)

S8 Fig 3’-RACE analysis. The fragment of *OsmiR156h* precursor was amplified using the nested adaptor primer and specific primers for the first exon of LOC_Os06g44034.
(TIF)

S9 Fig Sequence comparison of 3’-UTR of *OsmiR156h* precursor between WD44-*SDT* and WD44-*sdt*. Comparative DNA sequence of 3’-UTR was analyzed using 3’-RACE. The identical nucleotide sequences were showed by dark boxes, variant nucleotide sequence were shown by light boxes, and the polyA signal (“AATAAA”) was indicated by red boxes.
(TIF)

S10 Fig Comparison of the expression of *OsmiR156*-targeted *OsSPLs* between WD44-*SDT* and WD44-*sdt* plants. (**a**) Young tillers of 55-day-old plants. (**b**) Second topmost internodes of 80-day-old plants. (**c**) Flag leaf tissues of 80-day-old plants. The transcriptional levels of *OsSPLs* were determined by qRT-PCR. Transcript abundance relative to the level of the WD44-*SDT* plants set to be one. Data shown as mean ± SE (n = 3).
(TIF)

S11 Fig The phenotype of transgenic rice plants constitutively expressing *OsSPL14*. Mature plant appearance of the transgenic WD44-*SDT* plants carrying the *pActin*::*OsSPL14* construct. Scale bar: 20 cm.
(TIF)

S12 Fig The contrasting phenotype of NIL-*OsSPL14* ^*WFP*^/*SDT* and NIL-*OsSPL14* ^*WFP*^/*sdt*. Mature plant appearance of field-grown two NILs plants. Scale bar: 20 cm.
(TIF)

S13 Fig Schematic drawing of vectors used in this study bearing cDNA from *SDT*/*sdt* locus. All vectors have *pCAMBIA2300* backbone.
(TIF)

S1 Table Primers used for fine mapping and sequencing. (DOCX)

S2 Table Primers used for building DNA constructs. (DOC)

S3 Table Primers used for transcripts analysis. (DOC)

S4 Table Primers used for 3’-RACE analysis. (DOC)
