# Supplementary figures and images for: Regulation of OsmiR156h through Alternative Polyadenylation Improves Grain Yield in Rice
Source: PLoS One. 2015 May 8;10(5):e0126154. doi: 10.1371/journal.pone.0126154 (PMC4425700; doi:10.1371/journal.pone.0126154)

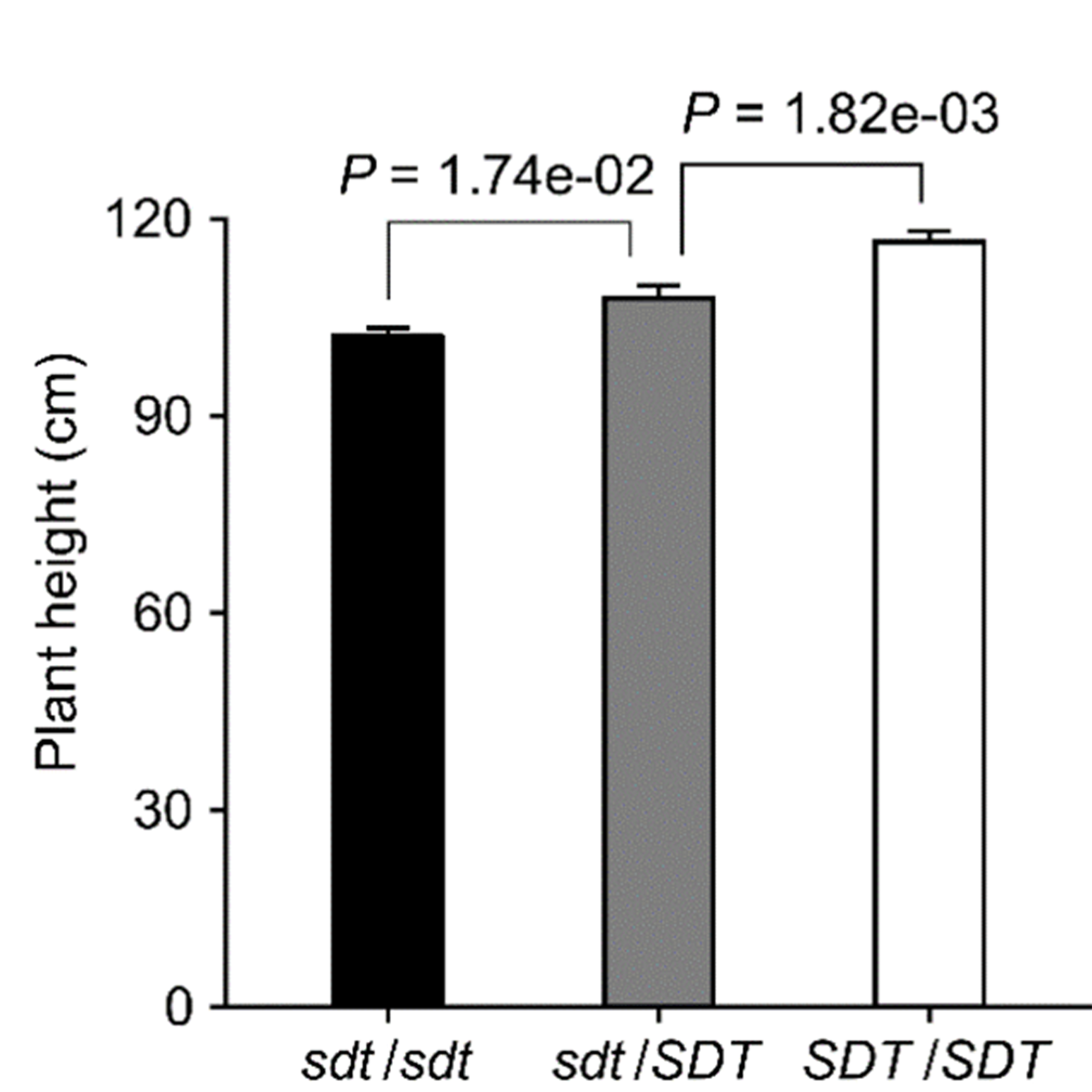

Supplement: S1 Fig — Segregation of the BC2F2 population derived from the backcross between the selected BC1F2 progeny carrying the sdt allele and 9311 plant. Comparisons of plant height among homozygotes of the sdt allele, heterozygotes of the sdt and the SDT allele, and homozygotes of the SDT allele. Data represented as mean ± SE (n = 30). (TIF) [file pone.0126154.s001.tif]

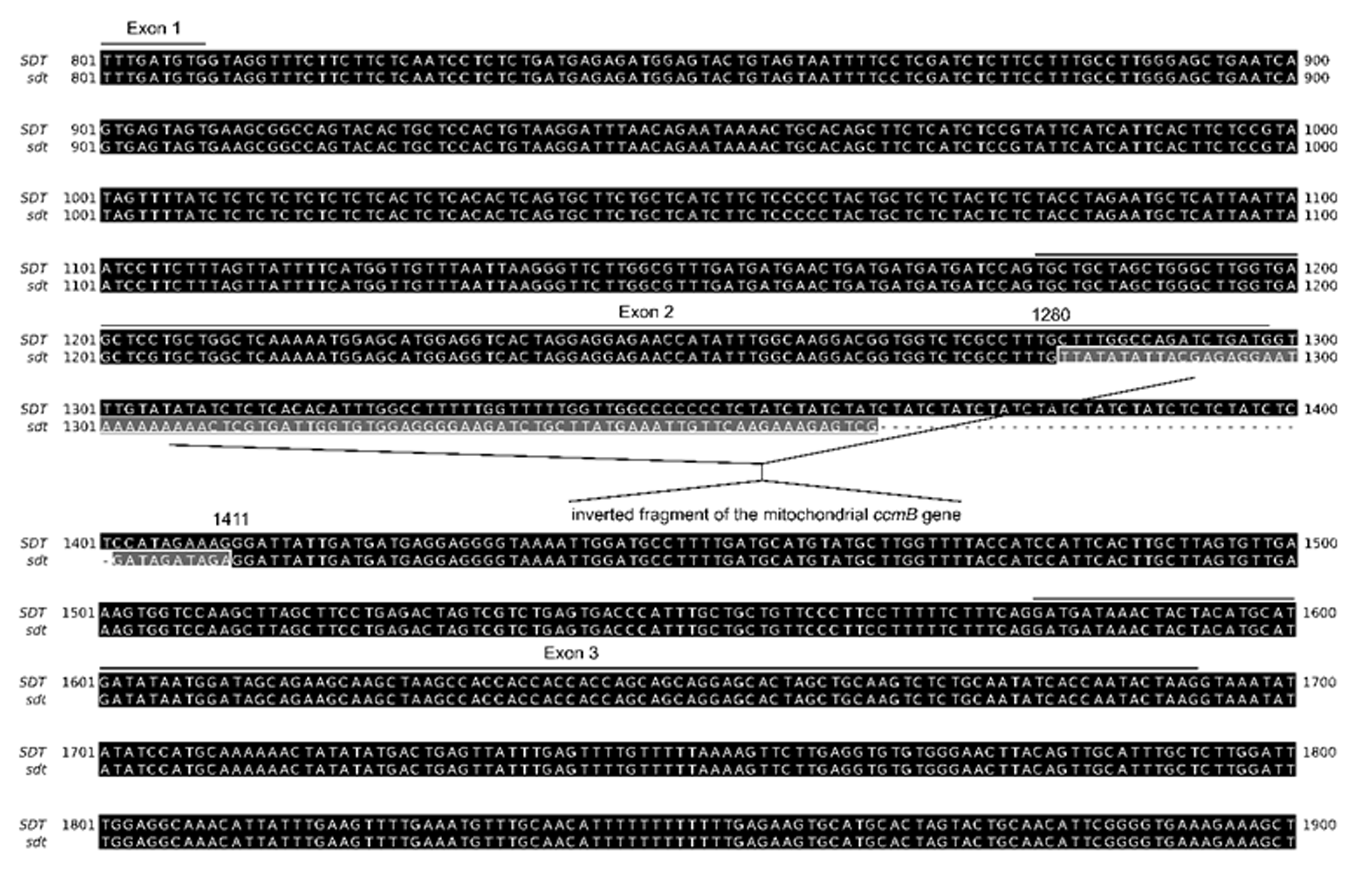

Supplement: S2 Fig — Allelic variation at the sdt locus, including a one-nucleotide substitution (g.1205C>G), a 131-bp deletion (g.1280_1401del) and an insertion of DNA fragment of the mitochondrial gene ccmB. The numbers indicate the position of the genomic sequence counted from the transcription start site of LOC_Os06g44034, and the lines above DNA sequences represent the location of the exons. (TIF) [file pone.0126154.s002.tif]

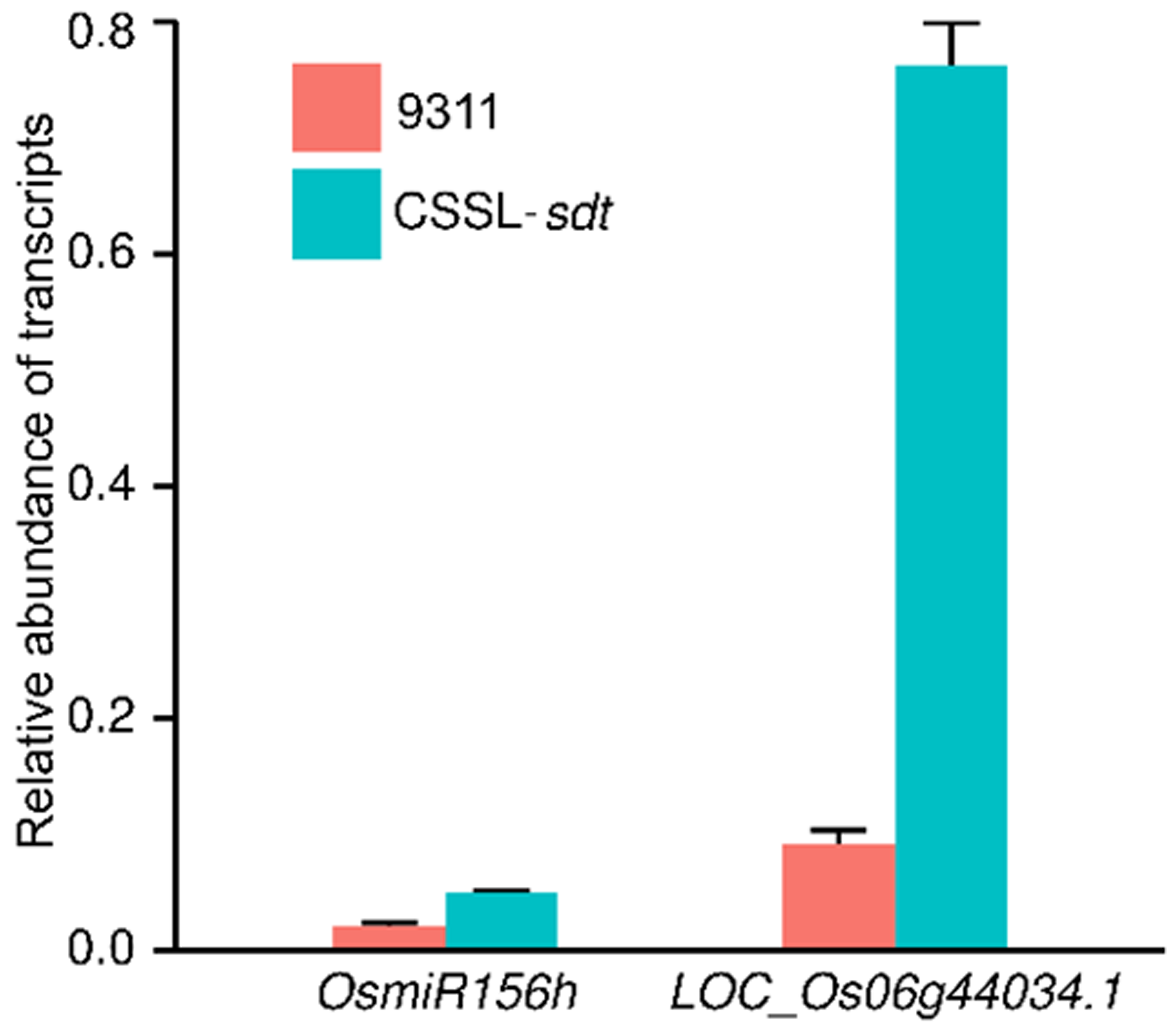

Supplement: S3 Fig — The transcriptional levels were determined by qRT-PCR using young leaf tissues. Expression levels are expressed as the relative copies per 1000 copies of rice actin3. Data given as mean ± SE (n = 3). (TIF) [file pone.0126154.s003.tif]

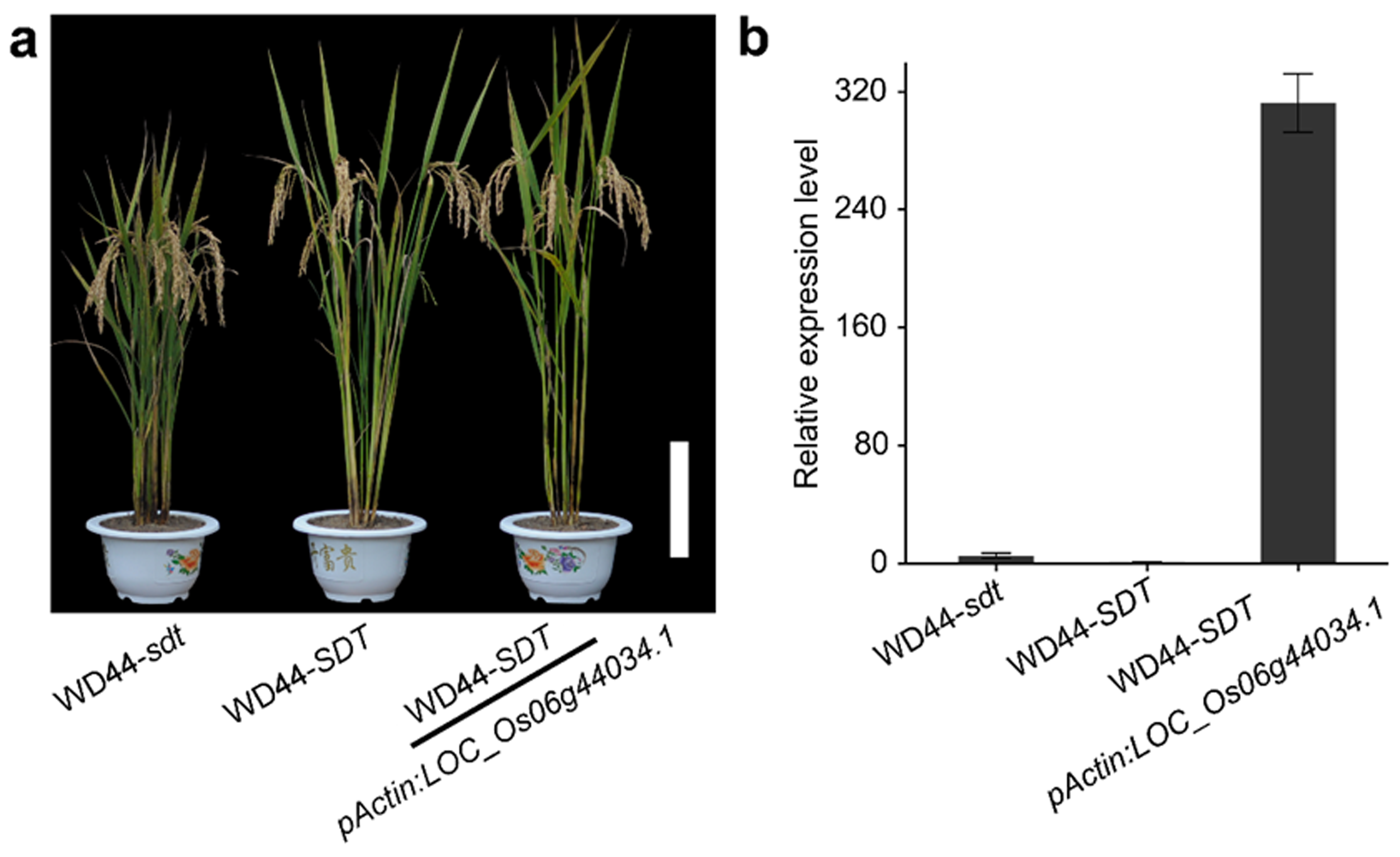

Supplement: S4 Fig — Mature plant appearance of the transgenic WD44-SDT plants overexpressing LOC_Os06g44034.1 under the control of rice Actin promoter. Scale bar: 20 cm. (TIF) [file pone.0126154.s004.tif]

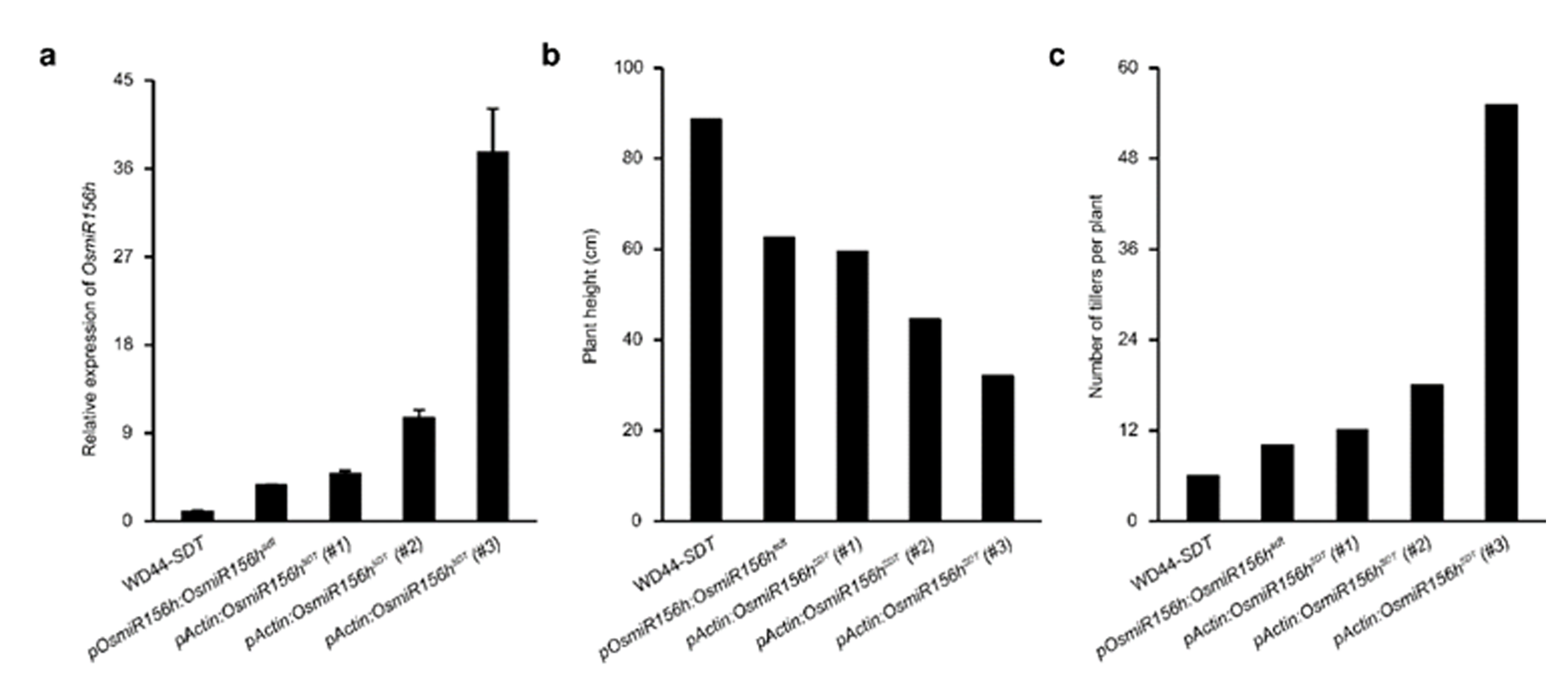

Supplement: S5 Fig — (a) The transcriptional levels of OsmiR156h were determined by qRT-PCR. Expression levels are expressed as the relative copies per 1000 copies of rice actin3. Data given as mean ± SE (n = 3). (b) Plant height. (c) Tiller numbers per plant. The transgenic plants were shown in Fig 1d. (TIF) [file pone.0126154.s005.tif]

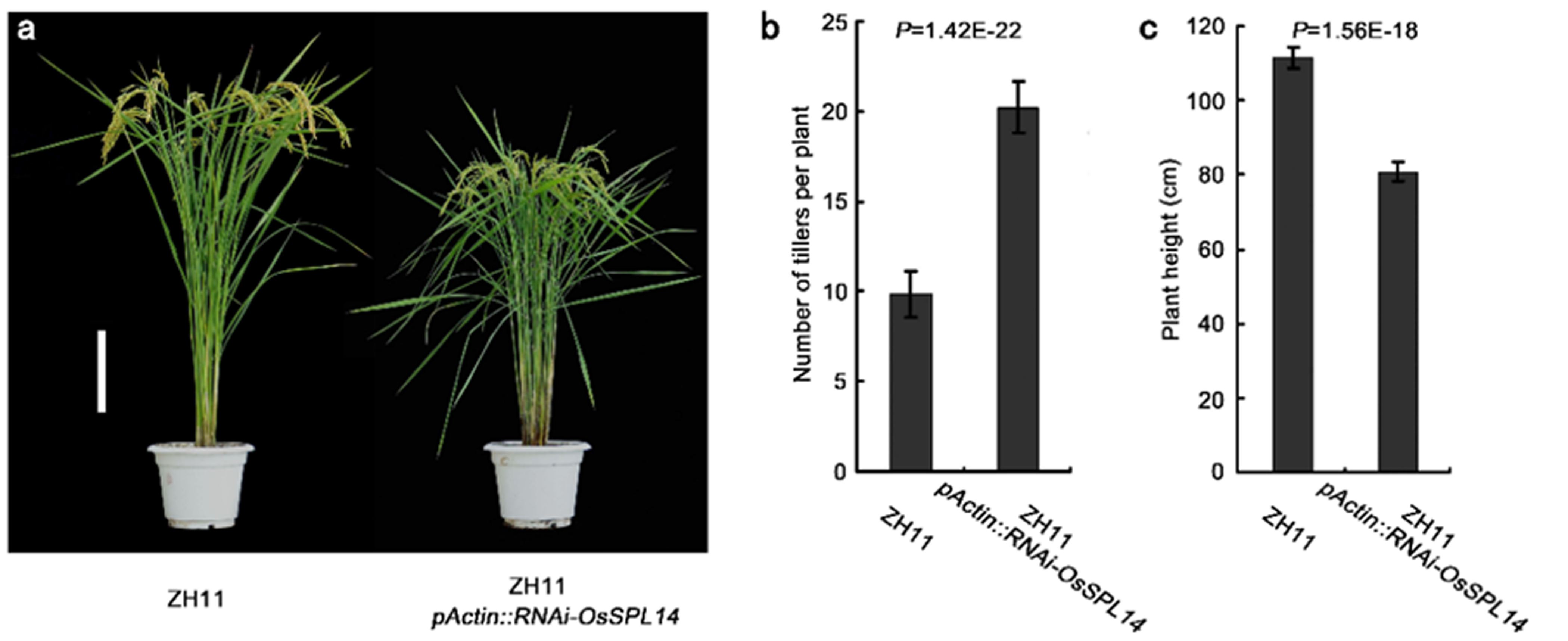

Supplement: S6 Fig — (a) Mature plant appearance. Scale bar: 20 cm. (b) Plant height. (c) Tiller numbers per plant. Data given as mean ± SE (n = 10). A Student’s t-test was used to generate the P values. (TIF) [file pone.0126154.s006.tif]

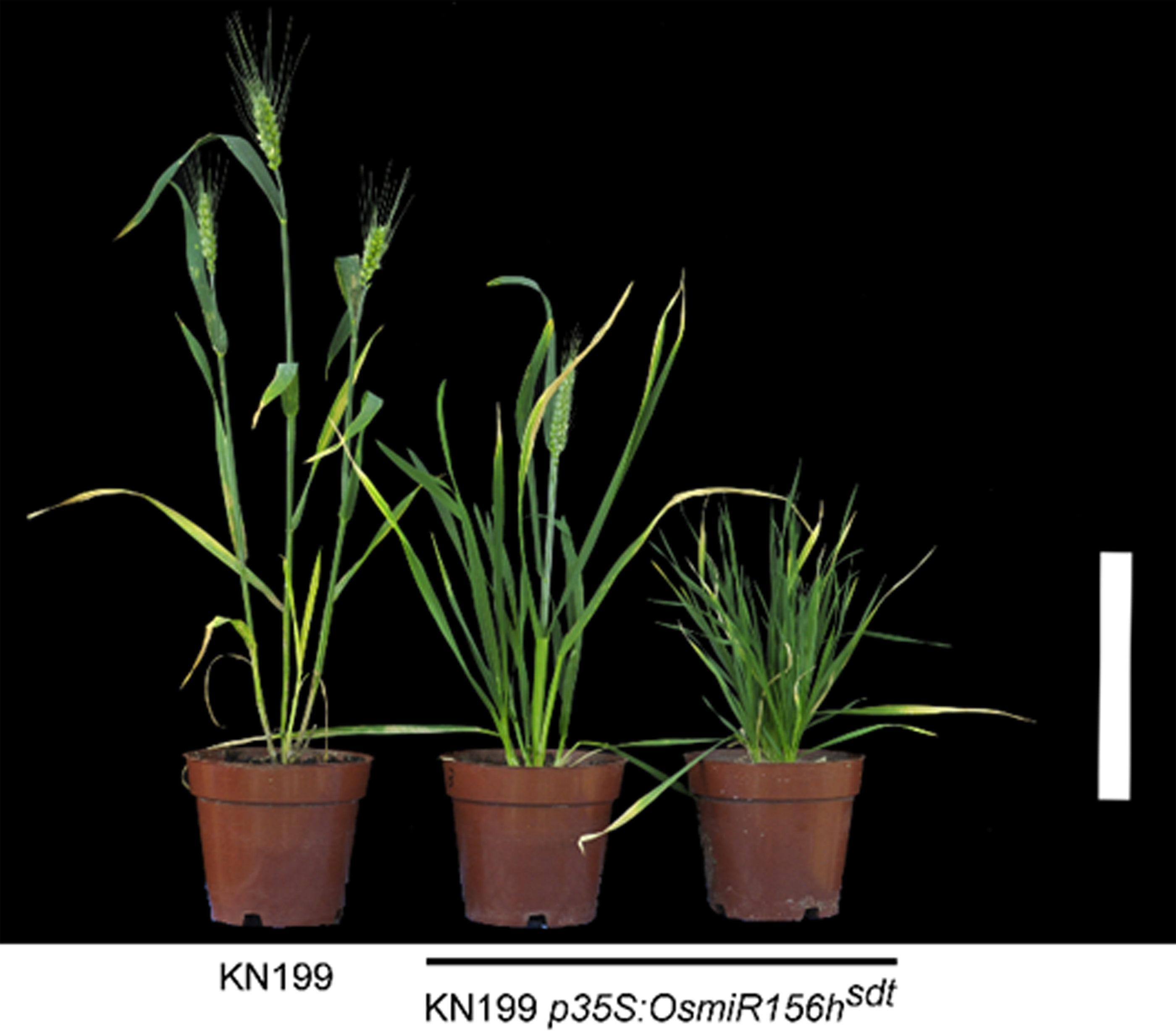

Supplement: S7 Fig — A winter wheat variety KN199 was used to generate the transgenic plants carrying the p35S::OsmiR156h sdt construct. Scale bar: 20 cm. (TIF) [file pone.0126154.s007.tif]

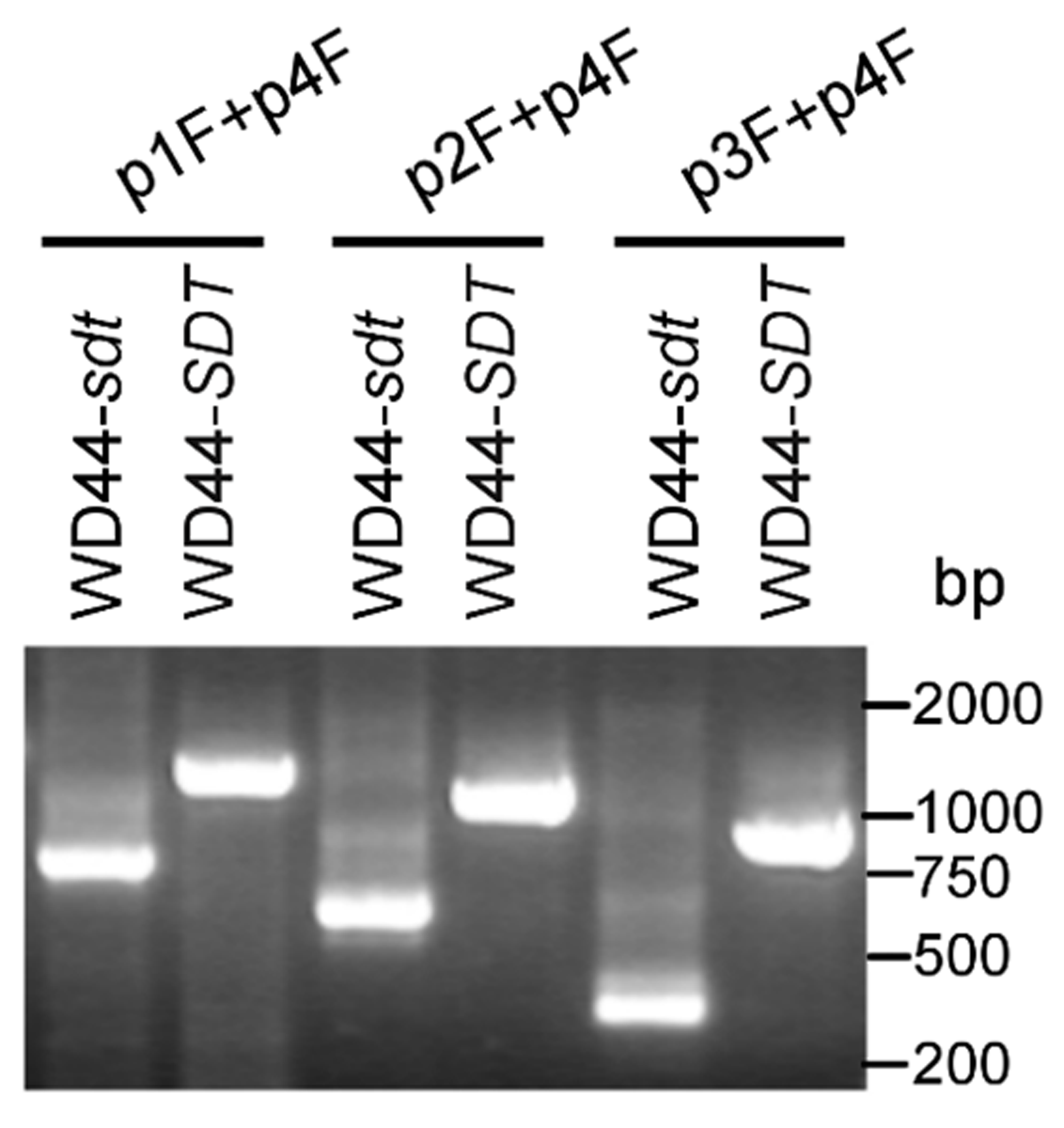

Supplement: S8 Fig — The fragment of OsmiR156h precursor was amplified using the nested adaptor primer and specific primers for the first exon of LOC_Os06g44034. (TIF) [file pone.0126154.s008.tif]

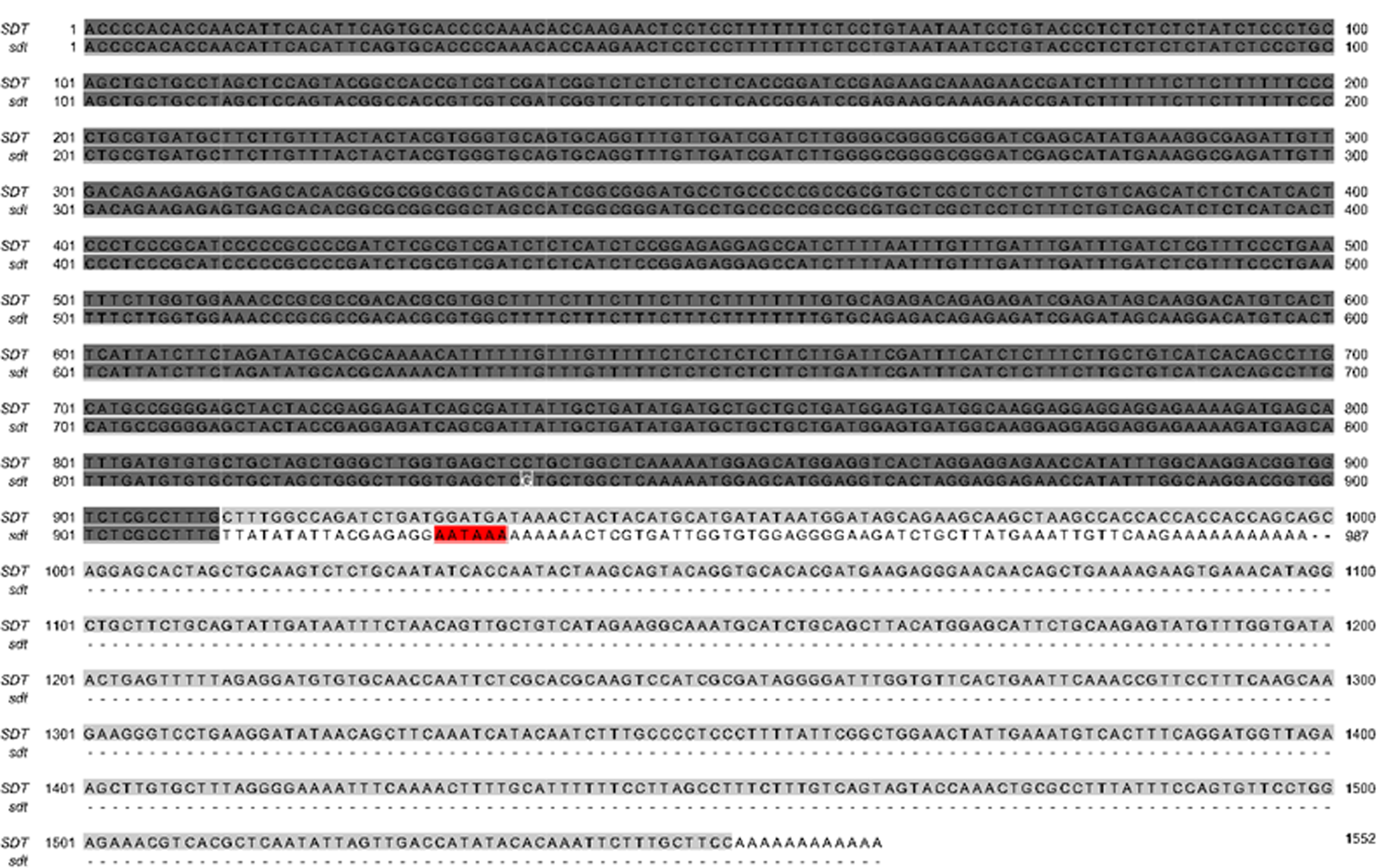

Supplement: S9 Fig — Comparative DNA sequence of 3’-UTR was analyzed using 3’-RACE. The identical nucleotide sequences were showed by dark boxes, variant nucleotide sequence were shown by light boxes, and the polyA signal (“AATAAA”) was indicated by red boxes. (TIF) [file pone.0126154.s009.tif]

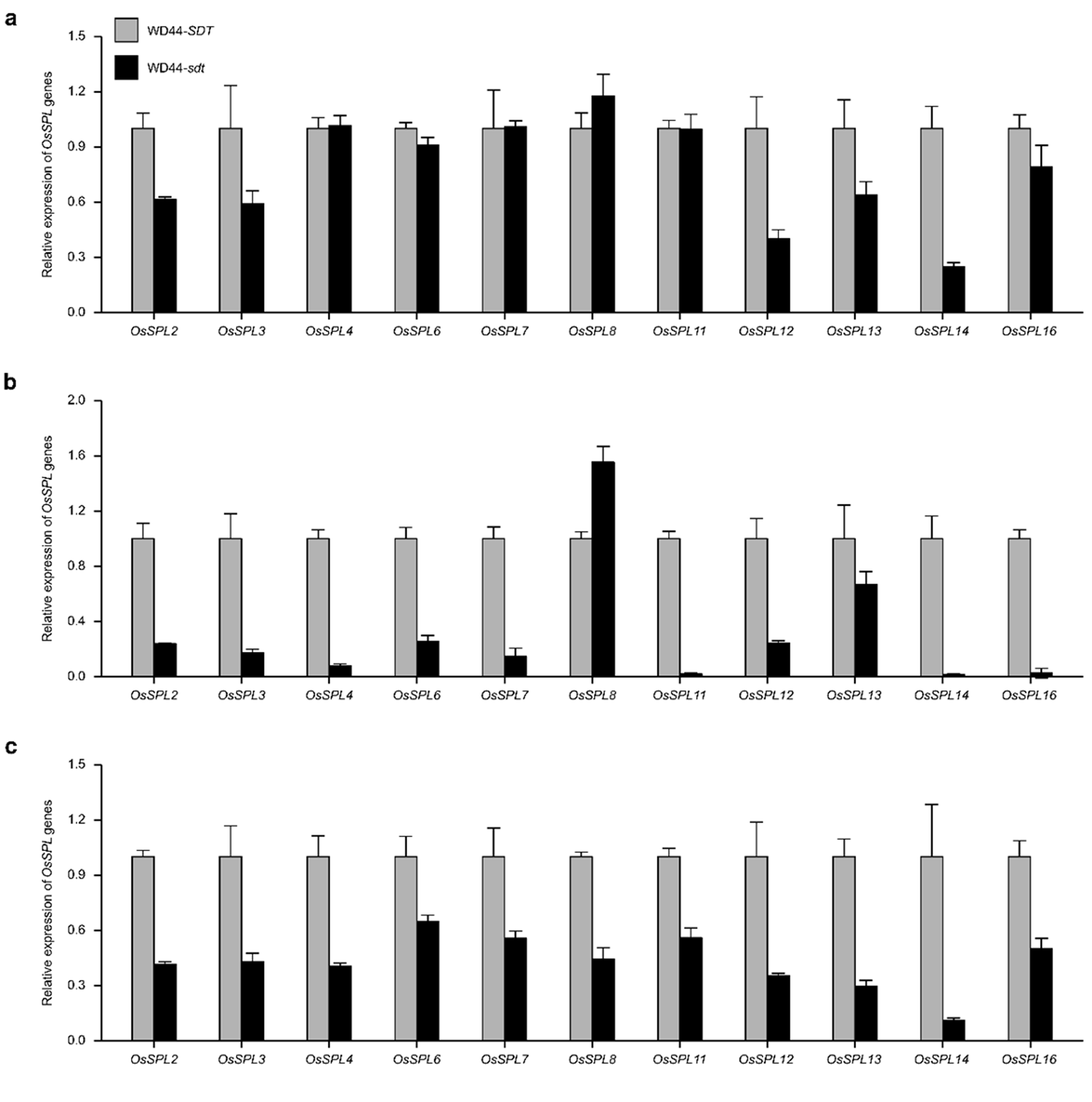

Supplement: S10 Fig — (a) Young tillers of 55-day-old plants. (b) Second topmost internodes of 80-day-old plants. (c) Flag leaf tissues of 80-day-old plants. The transcriptional levels of OsSPLs were determined by qRT-PCR. Transcript abundance relative to the level of the WD44-SDT plants set to be one. Data shown as mean ± SE (n = 3). (TIF) [file pone.0126154.s010.tif]

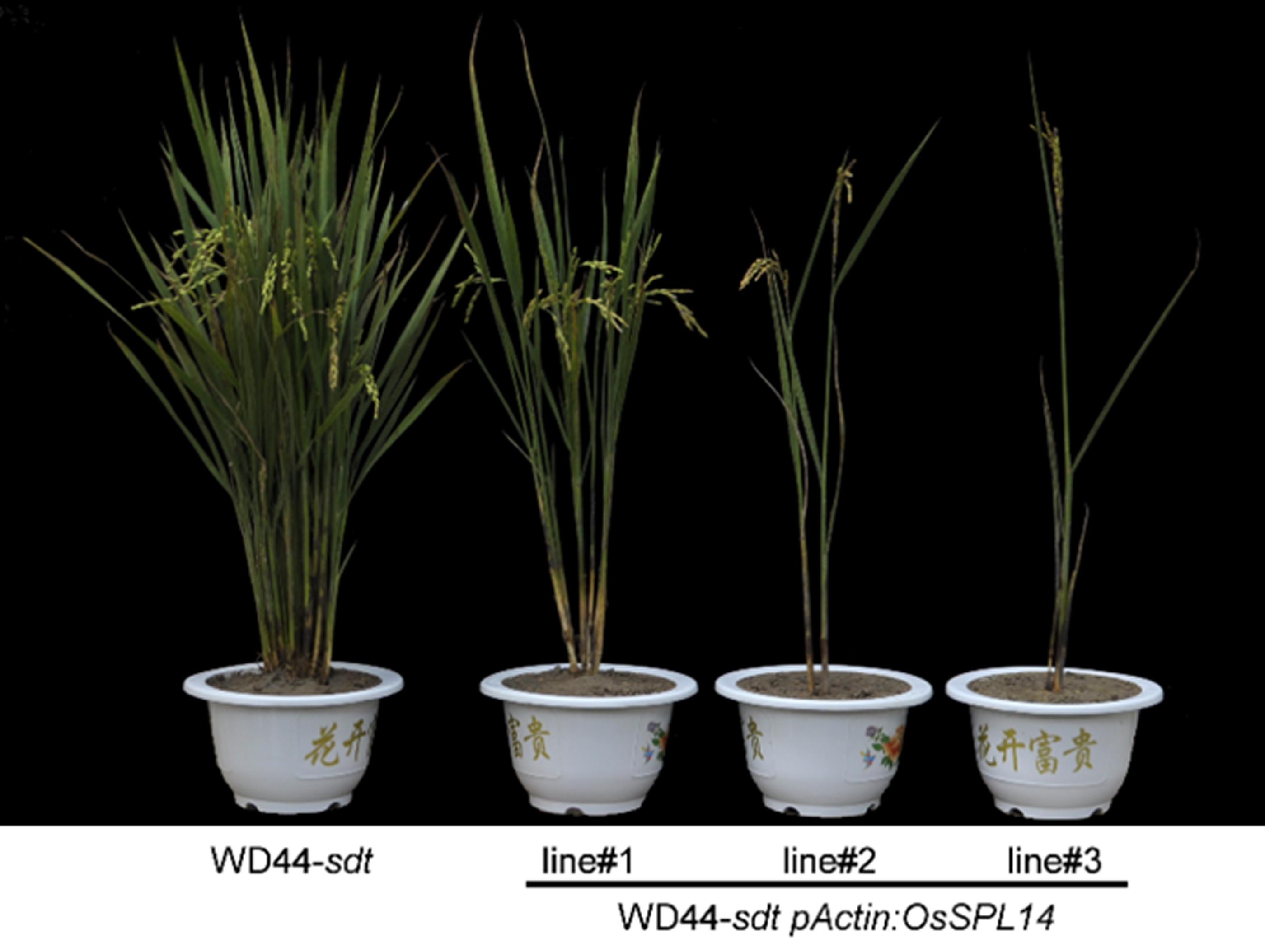

Supplement: S11 Fig — Mature plant appearance of the transgenic WD44-SDT plants carrying the pActin::OsSPL14 construct. Scale bar: 20 cm. (TIF) [file pone.0126154.s011.tif]

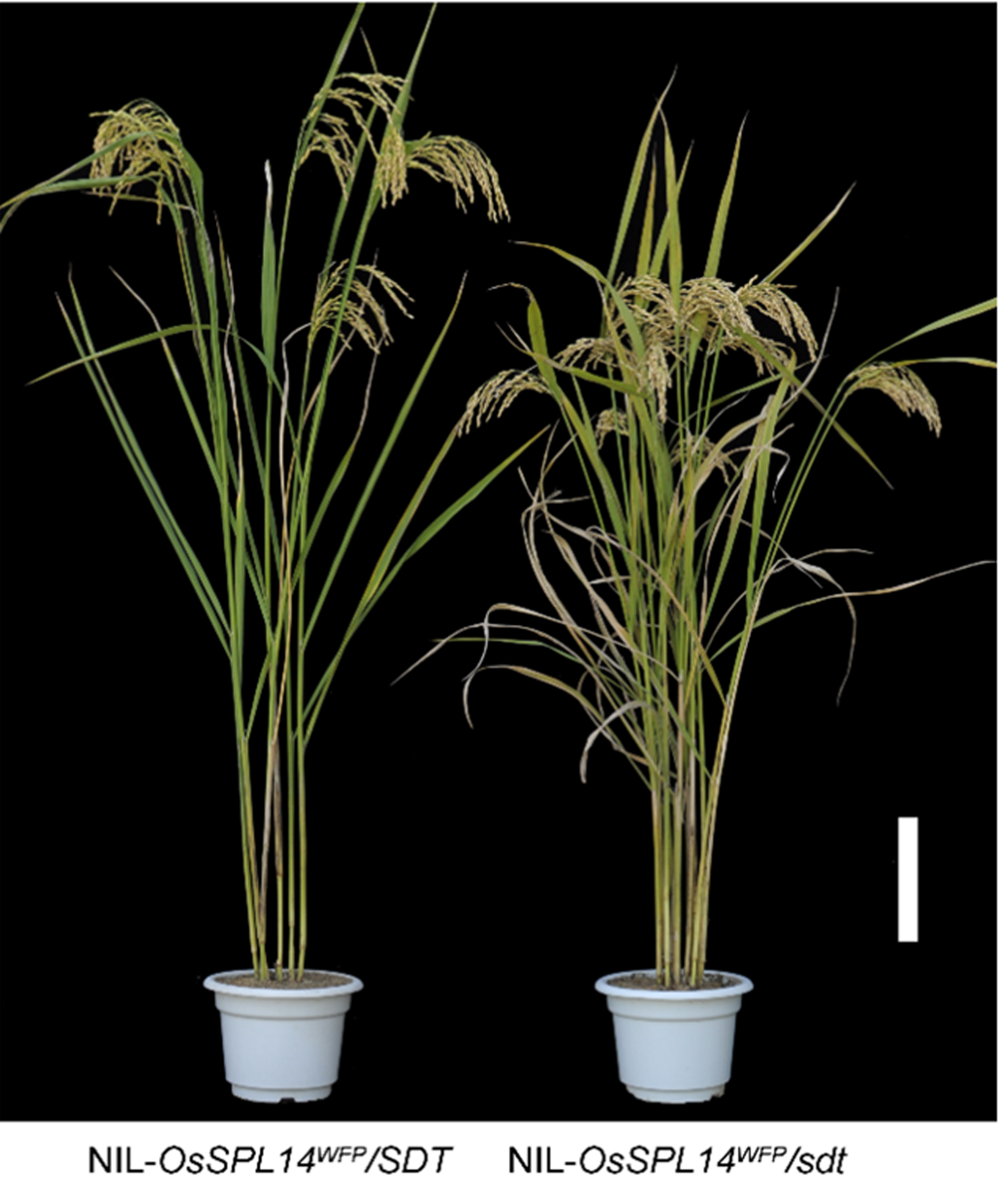

Supplement: S12 Fig — Mature plant appearance of field-grown two NILs plants. Scale bar: 20 cm. (TIF) [file pone.0126154.s012.tif]

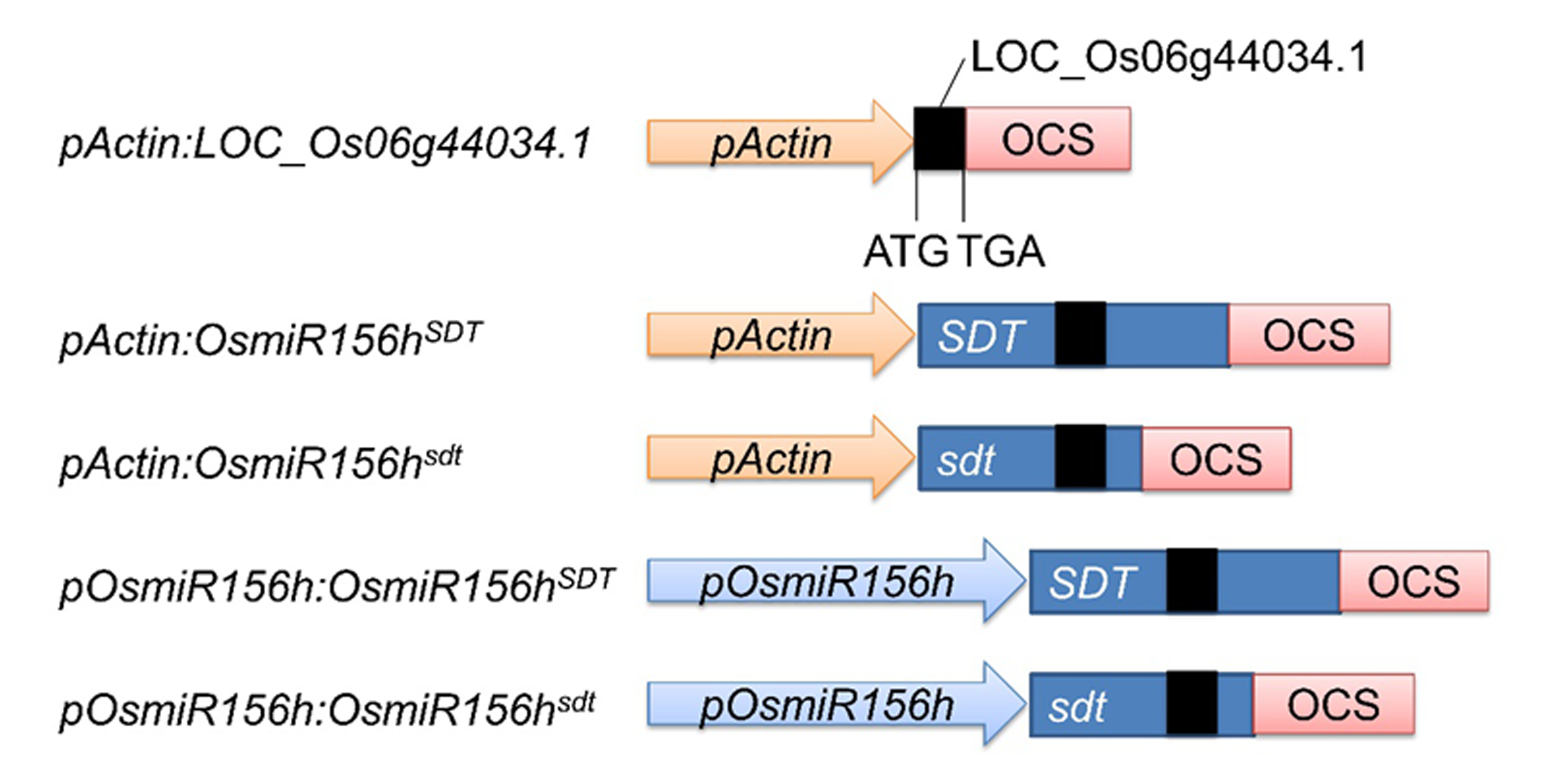

Supplement: S13 Fig — All vectors have pCAMBIA2300 backbone. (TIF) [file pone.0126154.s013.tif]
