# Supplementary material for: Regulation of OsmiR156h through Alternative Polyadenylation Improves Grain Yield in Rice
Source: PLoS One. 2015 May 8;10(5):e0126154. doi: 10.1371/journal.pone.0126154 (PMC4425700; doi:10.1371/journal.pone.0126154)
Supplement: S1 Table — (DOCX) [file pone.0126154.s014.docx]

**S1 Table. Primers used for fine mapping and sequencing**

| **Primers** | **Sequence (5'- 3’)** |
| --- | --- |
| 4008P3F | ttaaggccctttcgaacgta |
| 4008P3R | ccacagccagggtgtatttc |
| 3517P1F | ggcgtgtgtggagaaagaat |
| 3517P1R | gaccgtaatgcatgcaagtg |
| 3517P2F | aacctcgcatttggattttg |
| 3517P2R | ctgacctggtctccgtgatt |
| 3554p2F | gattttcgcgtcaacagagg |
| 3554p2R | cagccaaacatacgcacagt |
| 5453P1F | caagaagccaagaagcaagaa |
| 5453P1R | ggggaagactccagtgaagg |
| 5453P2F | gaaaacggagagacgcattt |
| 5453P2R | gcgagaaggaaaacgaatga |
| 5453P3F | ctagctgcagcacggactc |
| 5453P3R | cagtcagcgtgtgagagagg |
| 5453P4F | tcgatgaagtccctgtaccc |
| 5453P4R | tggatcttgttgctgctctg |
| 3565P1F | gctttttgtgatcgggtcat |
| 3565P1R | ccagcttcgtgttcatagca |
| 3579P1F | gacacgagcattgttttgct |
| 3579P1R | acactcggcattcctgattt |
| TAIL-156-2F | tccccctactgctctctactctctaccta |
| TAIL-156-AC-3F | acgatggactccagaggggttcttggcgtttgatgatgaactgatg |
| TAIL-156-4F | ctgctagctgggcttggtgagctcctgc |
| TAIL-156-8R | cccacacagggtctaaatacaccccaactaaactg |
| TAIL-156-AC-9R | acgatggactccagagcagcctatgcactgcactgaagcgtaaaaaagaatg |
| TAIL-156-10R | gaatgccaaacaacgcatgcagctttctttcaccccg |
| AC1 | acgatggactccagag |
