# Supplementary material for: Regulation of OsmiR156h through Alternative Polyadenylation Improves Grain Yield in Rice
Source: PLoS One. 2015 May 8;10(5):e0126154. doi: 10.1371/journal.pone.0126154 (PMC4425700; doi:10.1371/journal.pone.0126154)
Supplement: S2 Table — (DOC) [file pone.0126154.s015.doc]

**S2 Table. Primers used for building DNA constructs**

| **Primers** | **Sequence (5'-3')** |
| --- | --- |
| EcoR1-proSt | cggaattccttcccaaaaggaccctctc |
| Kpn1-mProEn | ggggtacctgtgcctaatataagctcacctctt |
| Xma1-cDNA-St | ccccccggggcaccccacaccaacatt |
| Sal1-cDNA-SDT-En | gcgtcgacggaagcaaagaatttgtgtatatgg |
| Sal1-cDNA-sdt-En | gcgtcgaccttgaacaatttcataagcagatctt |
| Xma1-PRO-St | ccccccgggatgtcacttcattatcttctagatatgca |
| Sal1-PRO-En | gcgtcgactcactccatcagcagcagc |
| Xma1-OsSPL14-St | ccccccgggatggagatggccagtggaggag |
| Xba1-OsSPL14-En | gctctagactacagagaccaatccatcg |
| SPLSPEF | ggactagtaagaacaaggggaagggcgtg |
| SPLBGLR | gaagatctaaaggggtttgcggcctcct |
| SPLXBAF | gctctagaaagaacaaggggaagggcgtg |
| SPLBAMR | cgggatccaaaggggtttgcggcctcct |
