# Supplementary material for: Regulation of OsmiR156h through Alternative Polyadenylation Improves Grain Yield in Rice
Source: PLoS One. 2015 May 8;10(5):e0126154. doi: 10.1371/journal.pone.0126154 (PMC4425700; doi:10.1371/journal.pone.0126154)
Supplement: S3 Table — (DOC) [file pone.0126154.s016.doc]

**S3 Table. Primers used for transcripts analysis**

| **Primers** | **Sequence (5'-3')** |
| --- | --- |
| pre-miR156-F | tgacagaagagagtgagcacac |
| pre-miR156h-R | gatgctgacagaaagaggagc |
| OsSPL2F | cgtgttccaagagccgtacta |
| OsSPL2R | gcagtggtagtggcagatttt |
| OsSPL3F | ttggagcacagaagttgttga |
| OsSPL3R | ccgatgcctgctgattagata |
| OsSPL4F | cgaaagtcagctcaggaat |
| OsSPL4R | gcaagccatcactccgttc |
| OsSPL6F | caccttcaccccagaagactac |
| OsSPL6R | ccagcacactaagctctcgac |
| OsSPL7F | gtgctcgcggagtttgac |
| OsSPL7R | cttcccacgcccagctgaca |
| OsSPL8F | gaacgcaggcattggagac |
| OsSPL8R | gaataagcacgagccagacg |
| OsSPL11F | gacggaaaccacagacagatg |
| OsSPL11R | ggtcagtcaaaccccttgaac |
| OsSPL12F | atccagcctgtgccgtcgtg |
| OsSPL12R | caggacaattcgccccca |
| OsSPL13F | tctagctctctccaccctcct |
| OsSPL13R | acctctccacctggcacct |
| OsSPL14F | caagggttccaagcagcgtaa |
| OsSPL14R | caacacgatggattggtct |
| OsSPL16F | caccacacaagtgctcgactcc |
| OsSPL16R | tggcagaaaagaaacagaaaca |
