# Supplementary material for: Regulation of OsmiR156h through Alternative Polyadenylation Improves Grain Yield in Rice
Source: PLoS One. 2015 May 8;10(5):e0126154. doi: 10.1371/journal.pone.0126154 (PMC4425700; doi:10.1371/journal.pone.0126154)
Supplement: S4 Table — (DOC) [file pone.0126154.s017.doc]

**S4 Table. Primers used for 3’-RACE analysis**

| **Primers** | **Sequence (5'-3')** |
| --- | --- |
| p1F | cagtgcaggtttgttgatcg |
| p2F | atctcgcgtcgatctctcat |
| p3F | ctgtcatcacagccttgcat |
| p4F | aaatggagcatggaggtcac |
